# Medication-related osteonecrosis of the jaws (MRONJ) in cancer patients treated with denosumab VS. zoledronic acid: A systematic review and meta-analysis

**DOI:** 10.4317/medoral.23324

**Published:** 2020-04-09

**Authors:** Alvaro Limones, Luis Miguel Sáez-Alcaide, Santiago Angulo Díaz-Parreño, Alexandra Helm, Michael M. Bornstein, Pedro Molinero-Mourelle

**Affiliations:** 1 Department of Conservative Dentistry and Orofacial Prosthesis. Faculty of Dentistry, Complutense University of Madrid, Spain; 2 Department of Dentistry. Universidad San Pablo CEU, Madrid, Spain; 3 Department of Dental Clinical Specialties. Faculty of Dentistry, Complutense University of Madrid, Spain; 4 Department of Statistics and Applied Mathematics. San Pablo CEU University, Madrid, Spain; 5 Applied Oral Sciences and Community Dental Care. Faculty of Dentistry, The University of Hong Kong, Hong Kong SAR, China; 6 Department of Oral Health and Medicine, University Center for Dental Medicine Basel UZB, University of Basel, Basel, Switzerland

## Abstract

**Background:**

The aim of the present study was to analyse the incidence, risk ratio (RR) and prognoses of two types of medication-related osteonecrosis of the jaws (MRONJ): denosumab-related osteonecrosis of the jaws (DRONJ) and Bisphosphonate-Related Osteonecrosis of the Jaws (BRONJ) in cancer patients under treatment with denosumab or zoledronic acid (ZA).

**Material and Methods:**

An electronic and manual search was conducted for randomized controlled trials (RCTs) until May 2019. Assessment of the identified studies, risk of bias and data extraction were performed independently by two reviewers. The incidence of DRONJ and BRONJ and the RR to develop MRONJ were calculated at 1 year, 2 years and 3 years of exposure. It was also calculated the odds ratio (OR) of their respective prognoses. They were calculated normalizing the values of the individual studies to 1 year, 2 years or 3 years when necessary through robust regression models using a statistical program.

**Results:**

From 1.277 references identified, 8 RCTs were included, which comprised a total of 13.857 patients with a variety of neoplasms. The incidence of DRONJ in cancer patients under treatment with denosumab ranged from 0.5 to 2.1% after 1 year, 1.1 to 3.0% after 2 years, and 1.3 to 3.2% after 3 years of exposure. The incidence of BRONJ in cancer patients under treatment with ZA ranged from 0.4 to 1.6% after 1 year of exposure, 0.8 to 2.1% after 2 years, and 1.0 to 2.3% after 3 years of exposure. Statistically significant differences were found between denosumab and ZA in the risk of developing MRONJ after 1, 2 and 3 years of exposure. Nevertheless, there were no significant differences in terms of patient prognosis.

**Conclusions:**

Denosumab is associated with a significantly higher risk of developing MRONJ compared to ZA. Nevertheless, no differences were found in its prognoses.

** Key words:**Denosumab, zoledronic acid, bisphosphonate-associated osteonecrosis of the Jaws, medication-related osteonecrosis of the jaws, neoplasms.

## Introduction

The increasing aging population goes hand in hand with a growing prevalence of disabling disease along with the use of medication to prevent and treat metabolic bone diseases (1). The bone is the most common site for metastasis, mostly associated with malignant tumours of the breast (73%), prostate (68%) or lung (36%) (2). Bone metastases can cause skeletal-related events (SREs) such as pain, pathological fractures, hypercalcemia and spinal cord compression, requiring radiation and surgery. They are also linked to an overall increase in mortality.

In 2009, denosumab was approved by the Food and Drug Administration of the United States (FDA) and the European Medicines Agency (EMA) for the treatment and prevention of bone metastases. Numerous case reports and case series have been published since then (3-6). In 2014, the American Association of Oral and Maxillofacial Surgeons (AAOMS) changed the term “Bisphosphonate-Related Osteonecrosis of the Jaws” (BRONJ) to "Medication-Related Osteonecrosis of the Jaws" (MRONJ) (7), as it is not only triggered by bisphosphonates, but also by other antiresorptive and antiangiogenic drugs such as monoclonal antibodies (MABs), tyrosine kinase inhibitors (TKI), mammalian target of rapamycin inhibitors (mTORi), selective estrogen receptor modulators (SERMs) and immunosuppressants (8). MRONJ can be the cause of serious functional and masticatory disorders with an important influence on patient quality of life and may even result in death (9).

To date, the pathophysiology of MRONJ has not been fully elucidated. It is believed to be multifactorial, due to a decrease in physiological bone remodelling, inflammation, infection, inhibition of angiogenesis, and innate or acquired immunity dysfunction (10,11). However, there are two emerging theories on the aetiology behind MRONJ. The first one, named “inside-outside”, is based on the inhibition of osteoclastic activity, resulting in a decrease of bone turnover. Due to this, jaw microdamage is not repaired and may lead to bone tissue necrosis and then to bone exposure over time. The second theory, termed “outside-inside”, is based on a local depression of the immune system, leading to local infection or inflammation inducing osteonecrosis (12).

The use of denosumab is expected to increase in the near future, because of its favourable profile in terms of avoiding adverse effects and renal toxicity compared to zoledronic acid (ZA) in the treatment and prevention of SREs in patients with advanced solid tumours (13,14). Several meta-analyses have already reported the incidence of DRONJ (15,16). Nevertheless, several new randomized-controlled clinical trials have been published recently. Therefore, the aim of this updated systematic review and meta-analysis is to compare the incidence, risk ratio (RR) and prognoses of DRONJ and BRONJ in cancer patients under treatment with denosumab and ZA.

## Material and Methods

This review was focused on answering the following three PICO questions: “In cancer patients under treatment with denosumab or ZA, do exist differences in the incidence of BRONJ (due to ZA) and DRONJ? If so, “what is the RR of MRONJ in patients treated with denosumab compared to patients treated with ZA?” and “do exist differences in the prognosis of BRONJ (due to ZA) compared to DRONJ?"

1) Study type: randomized clinical trials (RCTs).

2) Population: adult patients (> 18 years old) who were diagnosed with a solid tumour or with bone metastasis.

3) Intervention: subcutaneous denosumab in 120 mg doses every 4 weeks.

4) Comparison: intravenous ZA in 4 mg doses every 4 weeks.

5) Outcome: the primary outcome was the incidence of denosumab and zoledronic acid-related MRONJ; the secondary outcome was the RR of DRONJ compared to ZA-related BRONJ; and the third outcome was the OR of their respective prognoses.

- Eligibility criteria

Only double-blinded, ZA-control randomized clinical trials (RCTs) including patients followed up for at least 8 months were selected. RCTs without ZA-control, and non-randomized controlled clinical trials, retrospective and prospective studies were excluded. All studies were limited to research in humans published in English, French and Spanish.

- Search strategy

An electronic search for articles published from 2003 onwards was performed by entering the combination of the following search terms and Boolean operators: ((Cancer [All Fields] OR Neoplasms [MeSH Terms]) AND (Denosumab [MeSH Terms]) AND (“Zoledronic Acid” [MeSH Terms]) AND (“Bisphosphonate-Associated Osteonecrosis of the Jaw” [MeSH Terms] OR Osteonecrosis [MeSH Terms] OR MRONJ [All Fields] OR DRONJ [All Fields] OR ONJ [All Fields] OR ARONJ [All Fields])). An additional manual search was performed in selected journals of the field: “Journal of Dental Research”, “Oral Oncology”, “Clinical Oral Implant Research”, “International Journal of Oral Science”, “Oral Diseases”, “Journal of Oral Pathology & Medicine”, “International Journal of Oral and Maxillofacial Surgery”, “Journal of Cranio-Maxillofacial Surgery”, “Journal of Oral and Maxillofacial Surgery”, “Oral Surgery Oral Medicine Oral Pathology Oral Radiology”, “Medicina Oral Patología Oral y Cirugía Oral” and “International Journal of Clinical Oncology”.

- Information sources

**Figure 1:**
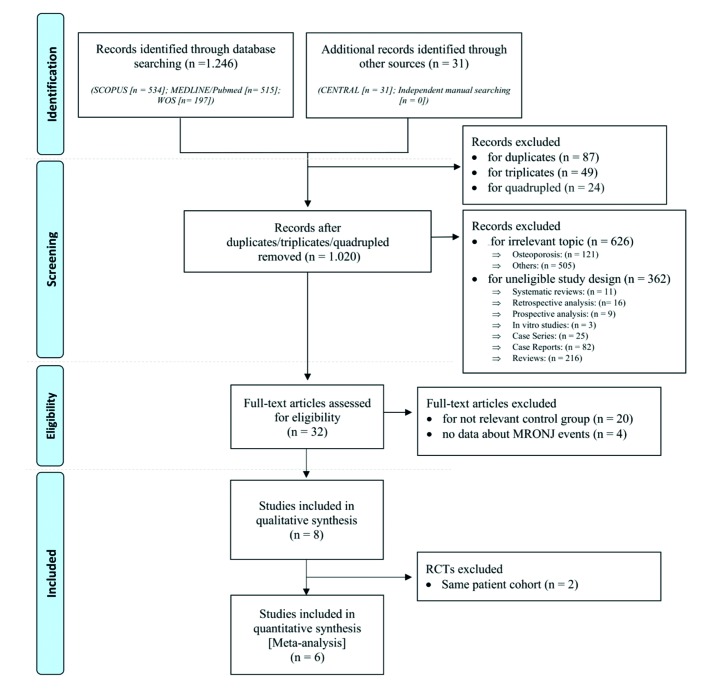
PRISMA flow chart for the present systematic review and meta-analysis.

The following five electronic databases were screened between January and May 2019: SCOPUS, MEDLINE (via OvidSP); Web of Science (WOS); the Central Registry of Controlled Clinical Trials of the Cochrane Library (CENTRAL), and the International Clinical Trials Registry Platform of the World Health Organization (WHO ICTRP) (Fig. 1).

- Study selection & Data collection and items

Study selection and data extraction were performed independently by two reviewers (A.L. & P.M.M.). In the case of disagreement on inclusion or exclusion, a consensus was reached by discussion with a third researcher (L.M.S.). The level of agreement between the reviewers was estimated using Cohen’s Kappa coefficient at title/abstract selection and at full-text selection. A Kappa value of more than 0.80 was considered as substantial agreement between the reviewers. The following items were entered into a Microsoft Excel spreadsheet (version 15.17, Microsoft Inc. 2015) (Table 1) (Table 2).

name of the first author, year of publication; country of origin; type of study; study population; number of participants; age; gender; follow-up; number of cases of MRONJ; cases of resolved MRONJ; type of drug (denosumab or ZA); dosage; frequency of administration, duration of drug administration, and route of administration.

**Table 1:**
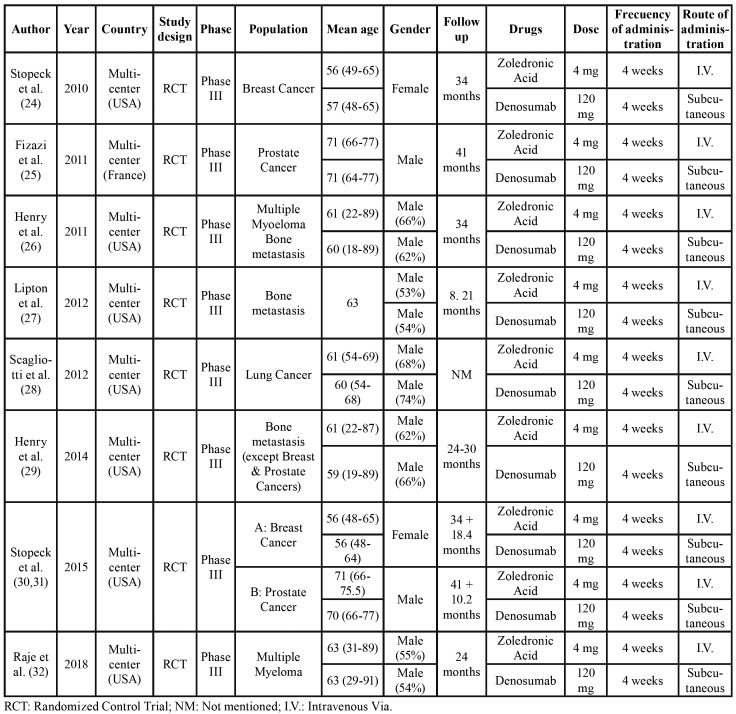
Characteristics of the included studies.

**Table 2:**
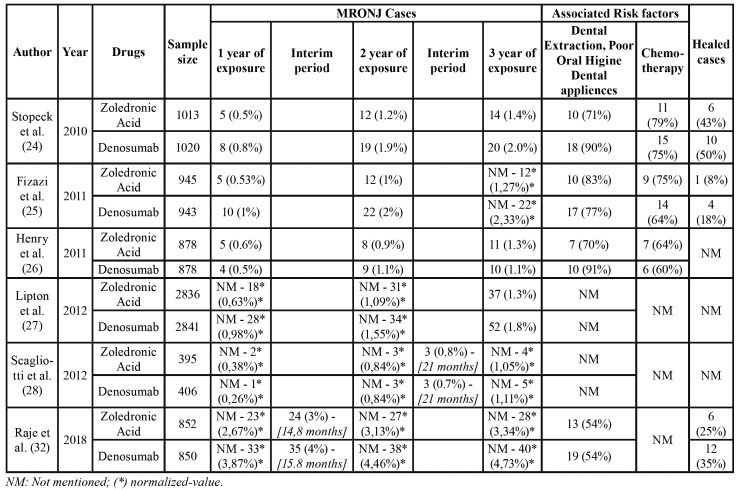
The incidence rate and prognosis of MRONJ reported by the included studies in the quantitative analysis and its normalization to 1 year, 2 years and 3 years of drug exposure.

- Statistical analysis

The incidence of MRONJ associated with denosumab and ZA was normalized when was necessary to 12, 24 and 36 months through a regression model using the statistical program IBM SPSS Statistics 25.0. The function S(t): exp (b0 + (b1/t)) was selected since it has the best fit with the course of the disease according to the incidence of each study and their follow-ups. The incidence was calculated analysing the MRONJ events among all participants involved in the included studies with a confidence interval (CI) of 95% using fixed or random-effect models depending on the heterogeneity of the included trials. Cochran’s Q test and I2 were used to determine statistical heterogeneity (18). If the I2 value was between 0 and 50% and *p-value* of the Q test was > 0.05, the level of heterogeneity was interpreted to be within accepTable limits, and therefore, a fixed-effect model would be applied.

The RR of MRONJ and the OR of the respective prognoses was calculated by comparing denosumab vs. ZA with the same meta-analytic methodology as described above. The analyses and forest plots were performed using Comprehensive Meta-Analysis version 3 software (Biostat, Englewood, NJ, USA). The overall quality of evidence for outcomes addressed by direct evidence (analyses with RR or OR) was rated using the Grades of Recommendations, Assessment, Development and Evaluation (GRADE) approach grouped into very low, low, moderate or high (19).

- Risk of bias of included studies

**Figure 2:**
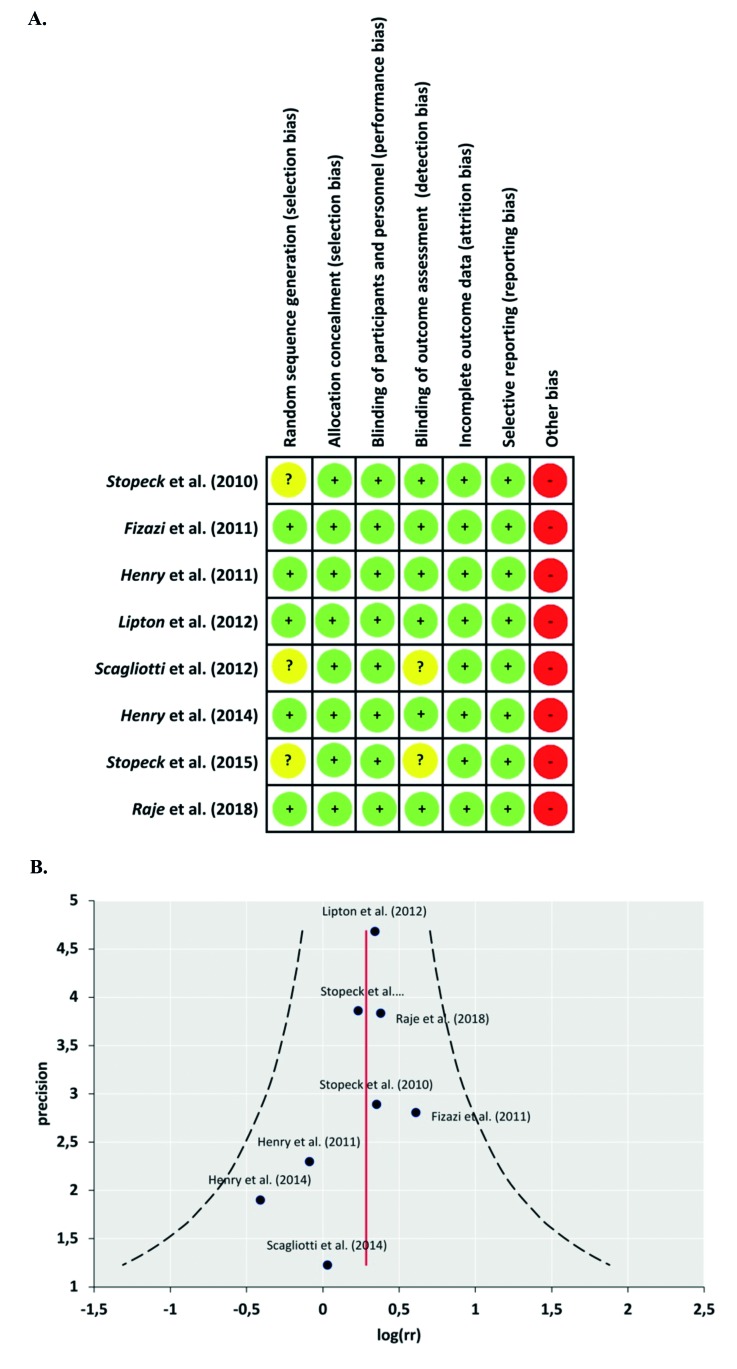
Assessment of the risk of bias. A) The summary of the Cochrane Collaboration’s tool for assessing risk of bias for the included studies. B) Evaluation of publication bias through Funnel plot.

The risk of bias for each study was determined using the Cochrane Collaboration Tool, described in the “Cochrane Handbook for Systematic Reviews of Interventions” version 5.1.0 (20). The articles were assessed according to 7 domains: selection bias, allocation bias, blinding of participants and staff, blinding of the outcome assessors, incomplete data, and selective notification of results. Furthermore, one category was added: conflict of interest, as it may be an important factor to take into consideration for pharmaceutically related studies. Each category was graded as low risk with a point, represented by [+[ in green, high risk with zero represented by [-] in red, and uncertain risk with half a point represented by [?] in yellow (Fig. 2) (21). Studies with 5 points or more have a lower risk of bias, whereas studies with less than five points exhibited a higher risk of bias (Table 3).

- Study quality

Publication bias was determined visually using funnel plots (22) using the statistical program Mix 2.0 (Fig. 2) (23).

**Table 3:**
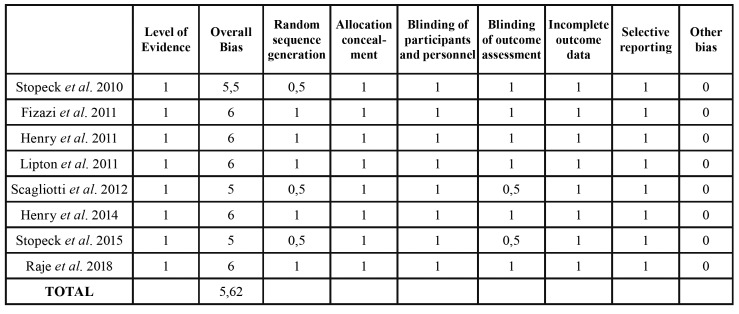
Tool for assessing risk of reporting biases.

## Results

- Study selection

The electronic database search yielded 1.277 references (515 in MEDLINE, 534 in SCOPUS, 197 in WOS, 31 in CENTRAL and 0 in WHO ICTRP) and duplicates (n = 87), triplicates (n = 49) and quadruplicates (n = 24) were removed. After screening by title and abstract, 32 papers were included for full-text assessment. No articles from the manual search were considered for inclusion. 20 articles did not comply with inclusion criteria due to a different control group, and 4 articles did not provide sufficient information on MRONJ. Eight RCTs met our inclusion criteria (Fig. 1) (24-32). Nevertheless, two papers were excluded from the quantitative analysis for reporting on the same patient cohort (29,30,31). A Kappa value of 0.874 was obtained for title/abstract selection, 0.841 for full-text selection and more than 0.90 for data extraction, indicating a high degree of inter-rater reliability.

- Characteristics of the studies

A summary of the general information of the included studies is shown in Table 1. The dosage, frequency and route of administration of denosumab and ZA was concurrent for all included studies. As some studies presented different follow-up times, it was necessary to normalize the incidences to specific moments of the follow-up period. A 12, 24 and 36-month normalization was selected as intermediate follow-up times. Table 2 shows a summary of MRONJ incidences reported by the studies and their normalizations when it was necessary to apply.

- Risk of bias of included studies

The risk of bias for each study was determined using the Cochrane Collaboration Tool.

The studies showed an average risk of bias score of 5.62 out of 7, indicating a low risk of bias (Fig. 2) (Table 3). However, all studies were funded by the pharmaceutical company that manufactures denosumab, which may result in some kind of bias.

- Publication bias

Funnel plot asymmetry should be used when there are at least 10 studies included in a meta-analysis. As seen in Fig. 2, there is moderate symmetry between studies. However, publication bias may not be detected since more studies are needed so that the power of evidence is strong enough to distinguish chance from real asymmetry (22).

- Results of the effect

**Figure 3:**
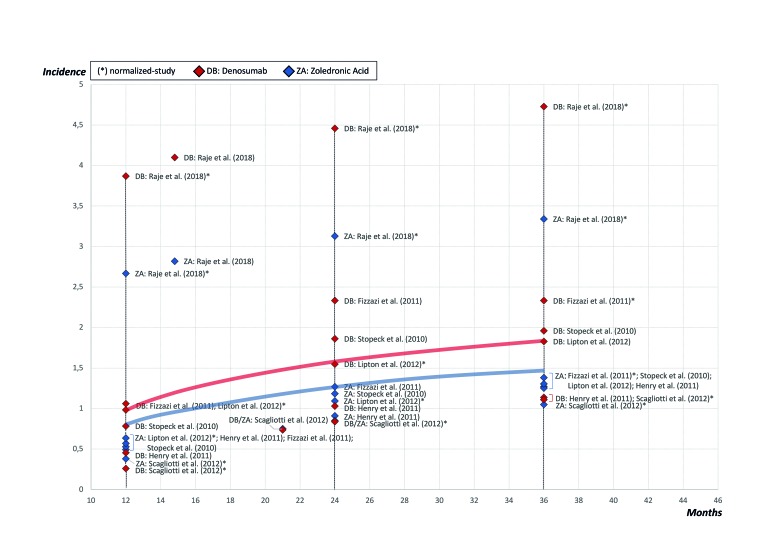
The summary of the normalized values of the individual studies and diagram of the course of MRONJ during the first 3 years.

The quantitative analysis included 6 RCTs comprising a total of 13.857 patients, of whom 6.938 belonged to the denosumab group and 6.919 to the ZA group, allowing for analyses with great statistical power. The estimates of the individual studies were normalized to 12-, 24- and 36-months using a regression model according to MRONJ incidence rates and the follow-up periods reported in the individual studies included. This allowed to draw a graph of the course of the disease that represents DRONJ and BRONJ incidences over time (Fig. 3).

When the pooled normalized incidence was calculated, statistical heterogeneity was detected for both groups. Therefore, a random-effects model was chosen. Nevertheless, a robust homogeneity was found when the RR or OR were calculated. A summary of the results of the meta-analysis is shown in Table 4. This analysis shows statistically significant differences between denosumab and ZA to develop MRONJ after 1 year (*P* = 0.030), 2 years (*P* = 0.006) and 3 years of exposure (*P* = 0.007). Nevertheless, no differences were found between the prognosis of DRONJ cases compared to cases due to ZA (*P* = 0.163). The individual studies reported a favourable prognosis varying from 18% to 50% for cases due to denosumab and 8% to 43% for ZA-related cases, within their respective observation periods.

**Table 4:**
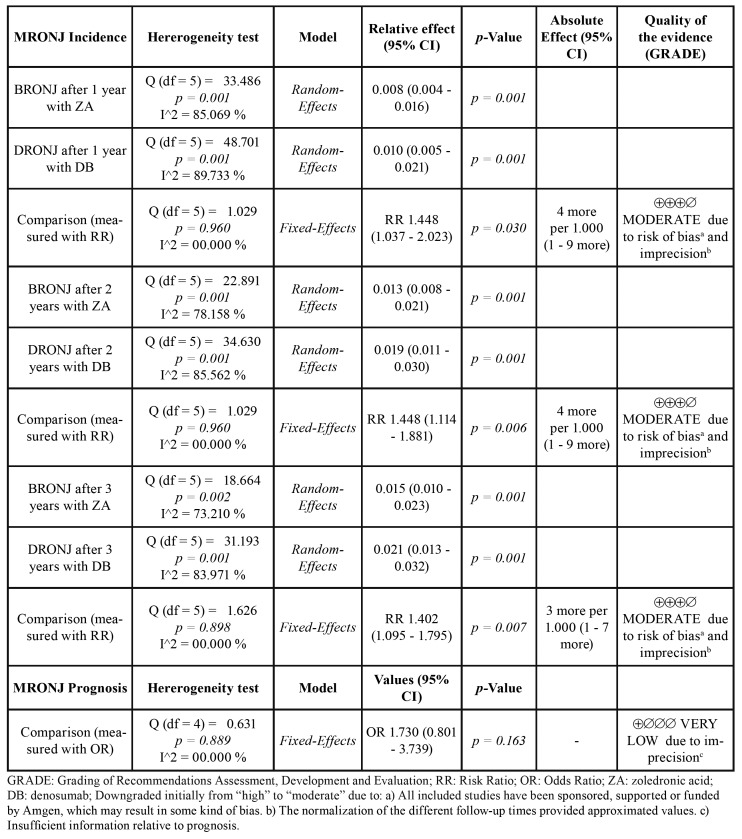
Meta-analysis results of the incidences and prognoses of MRONJ associated with zoledronic acid and Denosumab.

## Discussion

MRONJ is an uncommon but emerging complication of antiresorptive and antiangiogenic therapy. This updated systematic review and meta-analysis, which to our knowledge, includes all published randomized-controlled clinical trials to date comparing denosumab with ZA as control, aims to determine the incidence of MRONJ due to denosumab and ZA, the RR of developing MRONJ either due to denosumab or ZA, as well as the prognosis of DRONJ and ZA-related BRONJ.

This study has determined that the incidence of DRONJ in cancer patients under treatment with denosumab ranged from 0.5 to 2.1% (50 to 210 cases per 10.000 patients) after 1 year, 1.1 to 3.0% (110 to 300 cases per 10.000 patients) after 2 years, and 1.3 to 3.2% (130 to 320 cases per 10.000 patients) after 3 years of exposure. After longer periods of exposure, Stopeck *et al*. reported DRONJ incidences of 6.9% (30,31). In this sense, as it can be seen in Fig. 3, the MRONJ incidence is expected to increase over the exposure time. The incidence of BRONJ in cancer patients under treatment with ZA ranged from 0.4 to 1.6% (40 to 160 cases per 10.000 patients) after 1 year, 0.8 to 2.1% (80 to 210 cases per 10.000 patients) after 2 years, and 1.0 to 2.3% (100 to 230 cases per 10.000 patients) after 3 years of exposure. Some authors, such as Ruggiero *et al*. reported that in cancer patients under treatment with ZA, the risk of developing BRONJ reaches 1% (100 cases per 10.000 patients) and in patients under treatment with denosumab the risk of developing DRONJ ranges from 0.7 to 1.9% (70 to 190 cases per 10.000 patients) (7). Others, in contrast to these results, have reported a higher MRONJ incidence linked to denosumab (10 %, *P* = 0.21) with a median follow-up time of 22.0 months, and also due to bisphosphonates (6.7%, *P* = 0.21) (33). This higher incidence could be associated with the fact that the study of Loyson *et al*. is specifically aimed at evaluating MRONJ cases, contrary to the studies included in this review that have other priority objectives.

Many clinicians switch therapy from bisphosphonates to denosumab for their patients due to its multiple advantages, such as superior prevention of SREs, subcutaneous administration instead of intravenous administration, and no dosage adjustment in case of renal insufficiency (33). Loyson *et al*. reported that the switch from bisphosphonates to denosumab can be considered as safe as having an equivalent exposure to denosumab from the start (33).

According to the results of this analysis, the use of denosumab is associated with a significantly higher risk of developing MRONJ compared to ZA at 1 year (RR 1.448 CI: 1.037-2.023), 2 years (RR 1.488 CI: 1.114-1.881) and 3 years of treatment (RR 1.402 CI: 1.095-1.795). Thus, clinicians should be aware of this risk, promoting preventive measures such as comprehensive oral examinations with appropriate radiographs, oral hygiene instructions, maintenance of good oral health, completion of necessary dental treatments before initiating antiresorptive therapy etc. (34). Similarly, Owosho *et al*. reported that denosumab was associated with an earlier occurrence of MRONJ compared to ZA and pamidronate (35). Stavropoulos *et al*. consider that in patients under high doses of antiresorptive therapy, implant placement, explantation or their presence per se may trigger MRONJ (36). Most authors reported that MRONJ was strongly linked to dental extractions (45%) or poor oral hygiene (between 54 and 93%), simultaneous chemotherapy (between 60 and 75%), or the use of removable dentures with mucosal support (23,25,29-31). All of the causes mentioned above result in bacteria being in contact with susceptible bone or also in a decrease of the immune system in its role of protecting the oral mucosa from a bacterial-contaminated ecosystem.

No statistically significant differences were found in the prognosis of MRONJ cases due to denosumab or ZA (*P* = 0.163). However, individual studies reported that DRONJ cases have a slightly higher tendency to resolve, ranging from 18 to 50% compared to ZA, which ranges from 8% to 43%. This might be related to the reversibility mechanism inherent to denosumab, which is not found in bisphosphonates in general.

This systematic review and meta-analysis present certain limitations, such as the inclusion of different types of tumours, subcutaneously administered denosumab against intravenous ZA, the normalization of the different follow-up times providing approximated values or that all included randomized trials received some kind of funding by the pharmaceutical company that manufactures denosumab. On the other hand, the strengths of this study lie in the strict inclusion criteria used for homogeneous study selection, in the level of evidence provided by the selected RCTs, the low risk of bias of the individual studies, and in the sample sizes that allow for analyses with great statistical power.

Future RCTs should be carried out in more detail, measuring the real incidence per year, as well as the years after the exposure to medication for more clarity. Specifying further details could be of great value, such as whether oral examinations were carried out at enrolment or periodically, the intake of other concomitant drugs, the stage of MRONJ cases, and the number of patients undergoing dental surgical procedures along with the reason for surgery.

## Conclusions

Based on the findings of the present study, the use of denosumab is associated with a significantly higher risk of developing MRONJ compared to ZA. Thus, clinicians should be aware of this risk, and adapt or plan for preventive measures such as thorough dental examinations and interventions prior to the initiation of medication, as well as oral health maintenance and avoidance of surgical procedures during active therapy.
